# Off Target, but Sequence-Specific, shRNA-Associated Trans-Activation of Promoter Reporters in Transient Transfection Assays

**DOI:** 10.1371/journal.pone.0167867

**Published:** 2016-12-15

**Authors:** Tomohiro Masuda, Jun Wan, Anitha Yerrabelli, Cindy Berlinicke, Alyssa Kallman, Jiang Qian, Donald J. Zack

**Affiliations:** 1 Department of Ophthalmology, Wilmer Institute, Johns Hopkins University School of Medicine, Baltimore, Maryland, United States of America; 2 Institute of Genetic Medicine, Johns Hopkins University School of Medicine, Baltimore, Maryland, United States of America; 3 Department of Molecular Biology and Genetics, Johns Hopkins University School of Medicine, Baltimore, Maryland, United States of America; 4 Department of Neuroscience, Johns Hopkins University School of Medicine, Baltimore, Maryland, United States of America; 5 Institut de la Vision, University Pierre and Marie Curie, Paris, France; University of Cologne, GERMANY

## Abstract

Transient transfection promoter reporter assays are commonly used in the study of transcriptional regulation, and can be used to define and characterize both cis-acting regulatory sequences and trans-acting factors. In the process of using a variety of reporter assays designed to study regulation of the *rhodopsin* (*rho*) promoter, we discovered that rhodopsin promoter-driven reporter expression could be activated by certain species of shRNA in a gene-target-independent but shRNA sequence-specific manner, suggesting involvement of a specific shRNA associated pathway. Interestingly, the shRNA-mediated increase of rhodopsin promoter activity was synergistically enhanced by the rhodopsin transcriptional regulators CRX and NRL. Additionally, the effect was cell line-dependent, suggesting that this pathway requires the expression of cell-type specific factors. Since microRNA (miRNA) and interferon response-mediated processes have been implicated in RNAi off-target phenomena, we performed miRNA and gene expression profiling on cells transfected with shRNAs that do target a specific gene but have varied effects on rho reporter expression in order to identify transcripts whose expression levels are associated with shRNA induced rhodopsin promoter reporter activity. We identified a total of 50 miRNA species, and by microarray analysis, 320 protein-coding genes, some of which were predicted targets of the identified differentially expressed miRNAs, whose expression was altered in the presence of shRNAs that stimulated rhodopsin-promoter activity in a non-gene-targeting manner. Consistent with earlier studies on shRNA off-target effects, a number of interferon response genes were among those identified to be upregulated. Taken together, our results confirm the importance of considering off-target effects when interpreting data from RNAi experiments and extend prior results by focusing on the importance of including multiple and carefully designed controls in the design and analysis of the effects of shRNA on transient transfection-based transcriptional assays.

## Introduction

Rhodopsin, the visual pigment of rod photoreceptors (PRs), is essential for normal retinal development and for visual function [1–3]. The mechanisms regulating rhodopsin expression have been extensively studied for a number of years, both as a model of photoreceptor gene expression in general and because expression of rhodopsin has implications for retinal disease [4–6]. A number of the studies that have characterized the rhodopsin promoter and the factors that contribute to its expression were performed using transient transfection reporter assays. Among the rhodopsin regulatory factors that have been characterized using such assays are the transcription factors (TFs) cone—rod homeobox (CRX) and neural retina leucine zipper (NRL). CRX enhances photoreceptor-specific gene expression in both rod and cone PRs [7–10]. NRL promotes PR precursor cells to the rod lineage by simultaneously activating rod-specific genes, such as rhodopsin, and suppressing cone-specific genes [11–13]. CRX and NRL physically interact with each other [14], and promoter reporter assays show that they work together to synergistically enhance rhodopsin promoter activity [7].

In addition to the identification and characterization of factors such as CRX and NRL that directly regulate rhodopsin expression, in recent years transient transfection promoter studies have also been used to explore how post-transcriptional modifications can modulate the rhodopsin-promoting activity of retinal transcription factors. Based on studies demonstrating an important effect of the E3 SUMO ligase PIAS3 on PR differentiation [15, 16], we wanted to explore the possible role of the related family member PIAS2 in regulating rhodopsin expression. To accomplish this we initiated a series of promoter reporter transient transfection assays in HEK293 cells using a reporter vector containing a bovine rhodopsin promoter sequence upstream of a secreted luciferase reporter gene (*Gaussia* Luciferase, GLuc). Since PIAS2 is endogenously expressed in HEK293 cells, while CRX and NRL are not, we transfected the cells with CRX and NRL expression vectors in combination with short hairpin RNAs (shRNAs) either designed to target PIAS2, or designed as controls, and examined the effect these shRNAs had on *rho* promoter activity.

Unexpectedly, we found that many of the PIAS2 and other shRNAs tested had potent effects on rhodopsin reporter activity regardless of whether they targeted a relevant gene. The apparently non-gene targeting shRNAs that did affect rhodopsin expression did so in a sequence-specific and CRX/NRL-dependent manner. Thus, although our original intention was to investigate biological functions of PIAS2 on rho promoter activity, what we found indicates that rhodopsin promoter reporter expression can be influenced by specific but off-target shRNA effect. Furthermore, we found that the observed off-target effects were cell line dependent. Gene and microRNA (miRNA) profiling was performed and provided clues to, but did not fully define, the mechanism of the observed shRNA-mediated effects. Although there have already been many reports describing the potential off-target effects of various forms of RNAi-mediated gene knockdown [17, 18], we hope that by highlighting the complex interactions we experienced in our rhodopsin studies we will help highlight the multi-faceted controls and care in experimental interpretation that should be considered when shRNA knock-down is incorporated into transient transfection-based promoter studies.

## Materials and Methods

### Cell culture

HEK293 and COS7 cells were cultured in Dulbecco's Modified Eagle Medium (DMEM) supplemented with 10% fetal bovine serum (FBS) (Thermo Fisher Scientific, Waltham, MA) and 2 mM L-glutamine. Y79 and WERI cells were cultured in RPMI1640 supplemented with 10% FBS, 2 mM L-glutamine, and 10 mM HEPES. All cell lines were grown at 37°C in 5% CO2.

### Plasmids

pCMV-Gluc control vector (designated as CMV-Gluc) was purchased from New England BioLabs (NEB, Ipswich, MA). A bovine Rhodopsin promoter (-321 to -27 bp relative to the transcription start site) was subcloned into the multiple cloning site of pGLuc-Basic vector (NEB), pGL2-Basic vector (Promega, Madison, MI), and mRFP (created by replacing the eGFP sequence of pEGFP-N1 vector with mRFP) to generate the reporter plasmids rho-Gluc, rho-Fluc, and rho-mRFP, respectively. A rat *Nefm* promoter (-424 to +26 bp) was subcloned into the multiple cloning site of pGLuc-Basic to create Nefm-Gluc. A human *BEST1* promoter (-154 to + 38 bp) was subcloned into the multiple cloning site of pGL2-Basic to create BEST1-Fluc. pLKO.1 vector containing shRNA and pLKO.1 empty vector were from Thermo Fisher Scientific. pLKO.1 vector containing non-targeting scrambled shRNA and eGFP were obtained from Addgene [19](Cambridge, MA) and GE Healthcare (Pittsburgh, PA), respectively. The CMV early enhancer/chicken β actin (CAG)-driven human CRX and mouse NRL expression vectors were provided by Seth Blackshaw (Johns Hopkins University School of Medicine). Mutated PIAS2 shRNA vectors were constructed by ligating synthesized DNA oligos into *Age*I and *EcoR*I sites of pLKO.1 empty vector. Briefly, synthesized antisense- and sense-DNA oligos were mixed and denatured at 95°C for 4 min in a heat block, incubated at 70°C for 10 min in a water bath, then heat turned off and the samples were allowed to reach room temperature. Ligation was performed with Mighty Mix (Takara, Shiga, Japan) DNA ligation kit. The sequences of the shRNAs used in this study are listed in Table 1.

**Table 1:** shRNAs used in this study.

Gene	Species	shRNA ID	shRNA cat#	shRNA (5–3)
*PIAS2*	Human	shPias2_48	TRCN0000013348	CCGGGCCATGTTATTACAGAGATTACTCGAGTAATCTCTGTAATAACATGGCTTTTT
*PIAS2*	Human	shPias2_49	TRCN0000013349	CCGGCCACAATCAAATCATCGGTTTCTCGAGAAACCGATGATTTGATTGTGGTTTTT
*PIAS2*	Human	shPias2_50	TRCN0000013350	CCGGGCAAGCAAGAAGAAAGTAGATCTCGAGATCTACTTTCTTCTTGCTTGCTTTTT
*PIAS2*	Human	shPias2_51	TRCN0000013351	CCGGGCTGCTATTCCGCCTTCATTACTCGAGTAATGAAGGCGGAATAGCAGCTTTTT
*PIAS2*	Human	shPias2_52	TRCN0000013352	CCGGCGAGTTTAGTTCAAAGCAGTACTCGAGTACTGCTTTGAACTAAACTCGTTTTT
*PIAS2* mutant		shPias2_mu1	Synthetic oligo	CCGGCCACAATCAAAGAAAGTAGATCTCGAGATCTACTTTCTTTGATTGTGGTTTTT
*PIAS2* mutant		shPias2_mu2	Synthetic oligo	CCGGGCAAGCAAGAATCATCGGTTTCTCGAGAAACCGATGATTCTTGCTTGCTTTTT
*PIAS2* mutant		shPias2_mu3	Synthetic oligo	CCGGGCAAGCAAGAAGAAAGTGTTTCTCGAGAAACACTTTCTTCTTGCTTGCTTTTT
*PIAS2* mutant		shPias2_mu4	Synthetic oligo	CCGGGCAAGCAAAAATCATCGGTTTCTCGAGAAACCGATGATTCTTGCTTGCTTTTT
*PIAS2* mutant		shPias2_mu5	Synthetic oligo	CCGGGCAAGCAATAATCATCGGTTTCTCGAGAAACCGATGATTATTGCTTGCTTTTT
*PIAS2* mutant		shPias2_mu6	Synthetic oligo	CCGGGCAAGCAACAATCATCGGTTTCTCGAGAAACCGATGATTGTTGCTTGCTTTTT
*PIAS2* mutant		shPias2_mu7	Synthetic oligo	CCGGGCAAGCAACAAGAAAGTAGATCTCGAGATCTACTTTCTTGTTGCTTGCTTTTT
eGFP		shEGFP	RHS4459 (GE Healthcare)	CCGGTACAACAGCCACAACGTCTATCTCGAGATAGACGTTGTGGCTGTTGTATTTTT
		shScrambled	Plasmid#1864 (Addgene)	CCGGCCTAAGGTTAAGTCGCCCTCGCTCGAGCGAGGGCGACTTAACCTTAGGTTTTT
*GAPDH*	Human	shGAPDH_25828	TRCN0000025828	CCGGGCTCATTTCCTGGTATGACAACTCGAGTTGTCATACCAGGAAATGAGCTTTTT
*GAPDH*	Human	shGAPDH_25830	TRCN0000025830	CCGGCCAGGTGGTCTCCTCTGACTTCTCGAGAAGTCAGAGGAGACCACCTGGTTTTT
*GAPDH*	Human	shGAPDH_25836	TRCN0000025836	CCGGTCCGGGAAACTGTGGCGTGATCTCGAGATCACGCCACAGTTTCCCGGATTTTT
*HDAC1*	Human	shHDAC1_4814	TRCN0000004814	CCGGCGTTCTTAACTTTGAACCATACTCGAGTATGGTTCAAAGTTAAGAACGTTTTT
*HDAC1*	Human	shHDAC1_4815	TRCN0000004815	CCGGCGGTGGTTACACCATTCGTAACTCGAGTTACGAATGGTGTAACCACCGTTTTT
*HDAC1*	Human	shHDAC1_4816	TRCN0000004816	CCGGGCCGGTCATGTCCAAAGTAATCTCGAGATTACTTTGGACATGACCGGCTTTTT
*HDAC1*	Human	shHDAC1_4817	TRCN0000004817	CCGGCCGCAAGAACTCTTCCAACTTCTCGAGAAGTTGGAAGAGTTCTTGCGGTTTTT
*HDAC2*	Human	shHDAC2_4820	TRCN0000004820	CCGGCCAGCGTTTGATGGACTCTTTCTCGAGAAAGAGTCCATCAAACGCTGGTTTTT
*PPP2CA*	Human	shRNA_2485	TRCN0000002485	CCGGGAGGGATATAACTGGTGCCATCTCGAGATGGCACCAGTTATATCCCTCTTTTT
*Hdac3*	Mouse	shHDAC3_39389	TRCN0000039389	CCGGCGTGGCTCTCTGAAACCTTAACTCGAGTTAAGGTTTCAGAGAGCCACGTTTTT
*Hdac3*	Mouse	shHDAC3_39391	TRCN0000039391	CCGGGTGTTGAATATGTCAAGAGTTCTCGAGAACTCTTGACATATTCAACACTTTTT
*Hdac3*	Mouse	shHDAC3_39393	TRCN0000039393	CCGGGAGTTCTATGATGGCGACCATCTCGAGATGGTCGCCATCATAGAACTCTTTTT
*Bcl2l11*	Mouse	shRNA_9692	TRCN0000009692	CCGGGTGACAGAGAAGGTGGACAATCTCGAGATTGTCCACCTTCTCTGTCACTTTTT
*Bcl2l11*	Mouse	shRNA_9693	TRCN0000009693	CCGGTCTCAGGAGGAACCTGAAGATCTCGAGATCTTCAGGTTCCTCCTGAGATTTTT
*Bcl2l11*	Mouse	shRNA_9694	TRCN0000009694	CCGGCCCGGAGATACGGATTGCACACTCGAGTGTGCAATCCGTATCTCCGGGTTTTT
*Bcl2l11*	Mouse	shRNA_9695	TRCN0000009695	CCGGAGCTTCCATACGACAGTCTCACTCGAGTGAGACTGTCGTATGGAAGCTTTTTT

### Transient transfection assay

Cells cultured in a 24-well plate were transfected with 100 ng reporter vector in combination with 200 ng shRNA and 100 ng each of the CRX and NRL expression vectors unless specifically stated using Lipofectamine 2000 (Thermo Fisher Scientific) according to the manufacturer’s instructions. Empty pcDNA3 plasmid was used to adjust the plasmid level so all transfections were done with 600ng total plasmid DNA. Twenty uLs of culture medium containing the secreted *Gaussia* luciferase was collected 1 and 2 days post-transfection and luciferase activity was assayed using the BioLux Gaussia luciferase assay kit (NEB). The rho-Fluc reporter was assayed in cell lysates collected 2 days post-transfection using the Luciferase Assay System (Promega). Luminescence from both reporters was measured using the FLUOstar OPTIMA (BMG Labtech, Cary, NC) plate reader and reported as relative light units (RLU). The number of cells expressing the rho-mRFP reporter and the signal intensity of the expressed fluorescent protein was measured by flow cytometry using the Accuri C6 flow cytometer (BD Biosciences, San Jose, CA) as described below (see Flow cytometry section).

### Quantitative real-time PCR

One microgram of total RNA, extracted from cells using RNeasy MiniPlus kit (Quiagen, Valencia, CA), was used for cDNA synthesis using superscript III polymerase (Thermo Fisher Scientific) with random hexamers. Twenty μL PCR reactions, containing 5 μL cDNA, 10 μL 2x iQ SYBR Green Supermix (Bio-Rad, Hercules, CA) and 0.5 μM primers (Table 2), were used in an iQ^™^5 Multicolor Real-Time PCR Detection System (Bio-Rad) with the following cycle parameters: 3 min denaturation at 95°C, 45 cycles of 10-second denaturation at 95°C, 30-second annealing at 60°C, and 30-second elongation at 72°C. Data were analyzed with Bio-Rad iQ5 Standard Edition V 2.1 program. The relative amount of the target cDNA was then normalized to GAPDH expression.

**Table 2:** PCR primers.

Gene	Species	Forward (5' - 3')	Reverse (5' - 3')
*CRX*	Human	TGTTTGCCAAGACCCAGTACCC	TGCTGTTTCTGCTGCTGTCGCT
*GAPDH*	Human	TAGCCAAATTCGTTGTCATACC	CTGACTTCAACAGCGACACC
*HIST1H2BK*	Human	CACCAGCGCTAAGTAAACTTGCCA	AGAGGCCAGCTTTAGCTTGTGGAA
*IFIT2*	Human	CCTCATCCCTTCAGCATCAAG	GTCCAATCTTTTGCCATACCAG
*NRL*	Human	TGCCTCCTTCACCCACCTTCA	GCACAGACATCGAGACCAGC
*PIAS2*	Human	AAATGGGATTGAACAGAAGCGCCC	ACATGGCTGATGTAAGCTGCCGTA
*PIAS3*	Human	ACTTCTAGCCAGCGGTTTGAGGAA	ATCACATTTGGCTCCTGGCAGAAC

### Cell quantitation

Two days post-transfection, HEK293 cell nuclei were stained with Hoechst 33342 (Thermo Fisher Scientific). Cell number was quantitated using a Cellomics VTI automated microscope (Thermo Fisher Scientific). The Cellomics Target Activation Image analysis application was used to analyze images of 60 fields in triplicate per condition taken at 20x magnification.

### Flow cytometry

Two days post-transfection, HEK293 cells were detached from the well with 0.05% Trypsin-EDTA (Thermo Fisher Scientific), centrifuged at 150 g for 5 min, and then resuspended in PBS. 26,145 ± 1,864 events cells/sample were analyzed. Fluorescence was measured using the 585/40 bandpass filter and the threshold for mRFP expression was set based on untransfected HEK293 cell population. Based on this threshold, the percentage of the mRFP expressing cells and the median value of the positive signal intensity were obtained. These two values were multiplied and the resulting value was calculated as the relative ratio to the value of the control sample.

### nCounter miRNA expression profiling

RNA from HEK293 cells transfected with the rho-Gluc promoter reporter, and the CRX and NRL expression vectors and either the shPAIS2_49, shPAIS2_50 or empty vector was collected 15, 24, and 48 hrs post-transfection using Trizol (Thermo Fisher Scientific), and miRNA expression was measured using the nCounter miRNA expression system (NanoString Technologies, Seattle, WA). To obtain miRNA expression profiles, raw counts were log-transformed (base 2) and a linear regression model on the raw data of positive spike-in RNA hybridization controls (POS_B~E) to obtain their linear response signals was applied, which was then used to estimate the efficiency of hybridization. Based on the positive control signal, we normalized the miRNA expression data in that

Ei=(Ri−B)/Slope

where *E*_*i*_ is normalized *i*th miRNA expression, *R*_*i*_ is the raw count for *i*th miRNA, *B* is the linear response signal of POS_B, and *slope* is the slope of linear response signals of positive controls. Finally, we performed global normalization based on the mean values of all miRNAs across all different conditions. We performed paired t-test across three time points (15, 24, and 48 hrs) for each miRNA between the samples which were transfected with the CRX and NRL expression vectors and the samples which were transfected with the CRX and NRL expression vectors and shPIAS2_49 or shPIAS2_50. A miRNA was determined as differentially expressed if its p-values are less than 0.05 and the absolute value of its fold change (FC) is larger than 1.1.

### Microarray

Total RNA was collected from HEK293 cells transfected with rho-Gluc and the CRX and NRL expression vectors and either with or without shPAIS2_49 at 15 and 48 hrs post-transfection using Trizol and was used for gene expression analysis using Affymetrix Mouse Exon 1.0 arrays. The output signals from the chip were normalized and summarized as gene expression profiles using the Partek Genomic Suite software (GC pre-background adjustment, RMA background correction, and quantile normalization). Then we performed paired t-tests across two time points (15 and 48 hrs) between the samples which were transfected with the CRX and NRL expression vectors and the samples which were transfected with the CRX and NRL expression vectors and shPIAS2_49. The differentially expressed genes were identified for p < 0.1 and the absolute value of linear fold change (FC) larger than 1.25.

## Results

### shRNAs can stimulate Rho-Gluc promoter reporter activity in a sequence-dependent but target-independent manner

**Fig 1:**
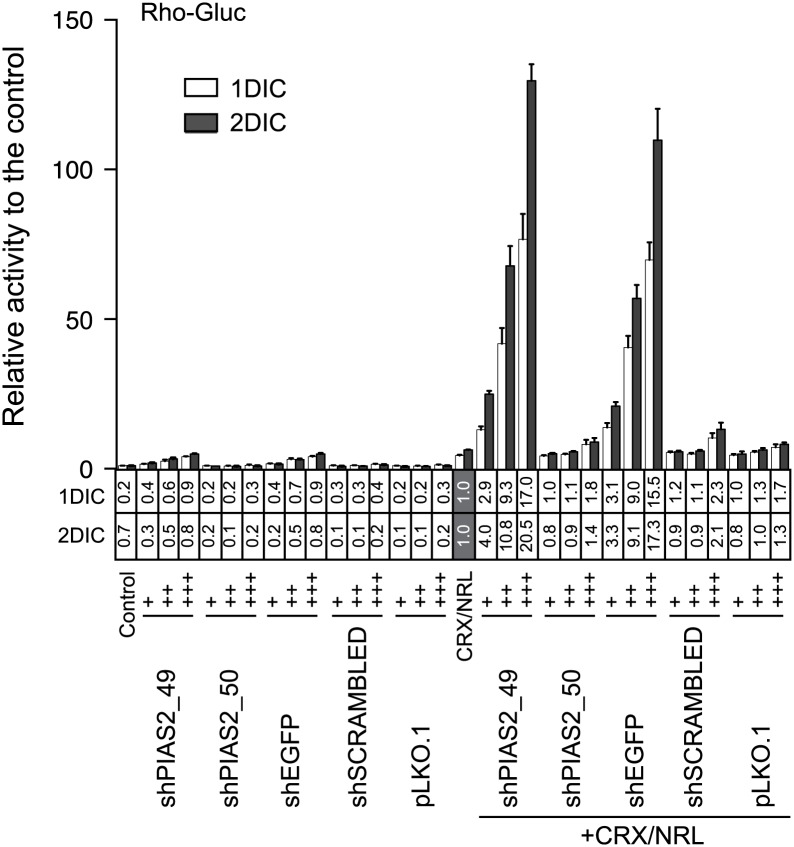
The effect of shRNA on the rho-Gluc activity is target-gene-independent. HEK293 cells were transfected with rho-Gluc reporter and the indicated shRNA with or without CRX and NRL expression vectors. '+', '++', and '+++' represent 8, 40, and 200 ng, respectively, of the indicated shRNA plasmid DNA. As a control, 500 ng of empty pcDNA3 with 100 ng reporter vector was transfected into HEK293 cells. *Gaussia* luciferase activity was measured in culture media one and two days after transfection. Relative luciferase activity to the control (bar graph) and the CRX/NRL transfected sample (in the column under the bar graph) are presented. Error bars are shown as SE.

With the goal of assessing the role of PIAS2 in photoreceptor gene expression, we performed shRNA-mediated *PIAS2* knockdown (KD) of endogenous *PIAS2* expression in HEK293 cells and measured the effect of the KD on rhodopsin (rho) promoter activity using a transiently transfected *Gaussia* luciferase reporter (rho-Gluc). A PIAS2 shRNA (shPAIS2_49) increased rho-Gluc activity by 2.6-fold at 2 days post-transfection (Fig 1), compared to the activity of an empty pcDNA3 control vector. When co-transfected with expression vectors encoding rho trans-activators CRX and NRL [7, 20], shPIAS2_49 synergistically enhanced rho-Gluc activity up to 88-fold; 8.6-fold higher than the activity induced by CRX and NRL alone. However, we found that transfection of shRNA for eGFP (shEGFP) also increased rho-Gluc activity by 4.9-fold, even though there is no specific target gene for shEGFP in HEK293 cells. Co-transfection of shEGFP with the CRX and NRL expression vectors synergistically enhanced rho-Gluc activity to a maximum of 110-fold by day 2; 17-fold higher than the activity induced by CRX and NRL. The trans-activation of the rhodopsin promoter reporter by an shRNA against eGFP suggested an off-target effect, possibly acting through the rhodopsin transcriptional regulatory network.

**Fig 2:**
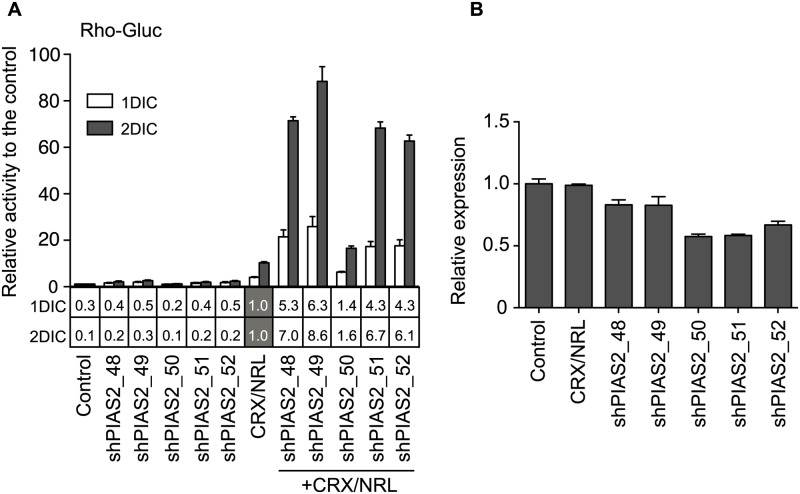
Rho-Gluc activity is synergistically enhanced by shPIAS2 in the presence of CRX and NRL regardless of the knockdown efficiency of endogenous *PIAS2*. (A) Transient co-transfection assay with rho-Gluc reporter. HEK293 cells were transfected with the reporter and one of five shPIAS2s with or without CRX and NRL expression vectors. Culture medium was collected one and two days after the transfection to measure *Gaussia* luciferase activity. Relative luciferase activity to the control (bar graph) and the CRX/NRL transfected sample (in the column under the bar graph) are presented. (B) Relative expression level of endogenous *PIAS2* mRNA. The expression level was examined by qPCR and normalized by that of *GAPDH*. Error bars are shown as +/- SE.

The strong trans-activation of the rhodopsin promoter reporter by a presumably “non-targeting” shRNA raised doubt as to whether the trans-activation of the rhodopsin promoter by PIAS2 shRNA was in fact due to a specific role for PIAS2 in regulation of the rhodopsin promoter. To more fully explore whether the impressive effects on rho reporter expression that we were observing were related to down-regulation of PIAS2, we examined the effect of five different PIAS2 shRNAs (shPIAS2_48–52), each designed to target a different region of the gene, on rho-Gluc activity. And simultaneously, we determined the relative knockdown of endogenous *PIAS2*, by qPCR, with each of the *PIAS2* shRNAs. The five different PIAS-targeting shRNAs increased rhodopsin expression between 1.1–2.6 fold, relative to control, at two days post transfection (Fig 2A). With the inclusion of CRX and NRL expression constructs, rho-Gluc activity induction increased to 16 to 88-fold; 1.6 to 8.6-fold higher than the activity induced by CRX and NRL alone. The effect that the five PIAS2 shRNAs had on endogenous *PIAS2* mRNA, as measured by qPCR, showed expression to be reduced between 17 to 43% (Fig 2B). However, there was no correlation between the *PIAS2* knockdown efficiency and the induction of rho-Gluc activity of these shPIAS2s (R^2^ = 0.49), further brining into question the original data that had suggested a role for PIAS2 in modulating rhodopsin promoter activity. We also tried to examine if there is a correlation between PIAS2 protein level and rho-Gluc activity, but PIAS2 expression was below detection level in HEK293 cells by Western blotting (data not shown).

While shPIAS2_49 and shEGFP enhanced rho-Gluc activity through a seemingly gene-target independent pathway, it is not a pathway that is engaged by all shRNAs. One shRNA designed to target PIAS2, shPIAS2_50 did not enhance rho-Gluc activity, and neither did the shScrambled or the empty pLKO.1 shRNA vector (when transfected with CRX/NRL, shPIAS_50 increased expression 1.6 fold, shScrambled 2.1 fold, and pLKO.1 1.3 fold).

### Rho-Gluc activating shRNAs do not alter cell density or reporter stability

**Fig 3:**
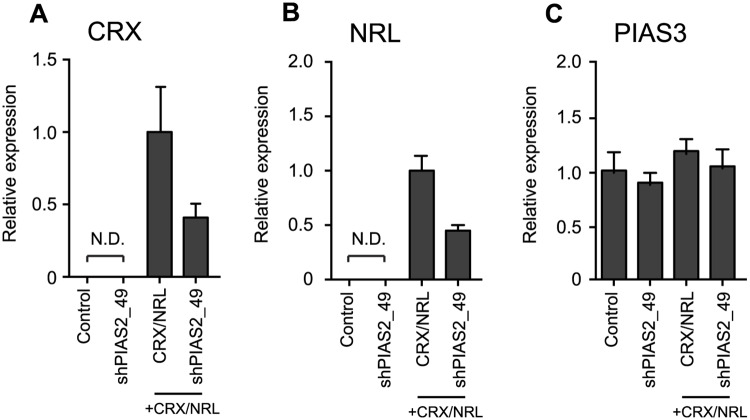
Relative mRNA expression level of Rhodopsin regulating factors in HEK293 cells. HEK293 cells were transfected with rho-Gluc reporter in combination with shPIAS2, CRX, and NRL expression vectors. Relative mRNA expression level of *CRX* (A), *NRL* (B), and *PIAS3* (C) quantified by qPCR at 2 days after the transfection. N.D. represents no data. Error bars are shown as SE.

The observed off-target effects of shRNAs on rho-Gluc activity could have been mediated by a variety of mechanisms, including modulation of rhodopsin transcription factor expression levels, cell viability or something specific to the reporter, such as affects on the reporter protein’s stability or activity. Since CRX and NRL are potent transcriptional activators of the rhodopsin gene [7, 20], if shRNA activates the expression of endogenous and/or exogenous CRX and NRL, the reporter activity may rise. Based on this hypothesis, we determined *CRX* and *NRL* mRNA expression levels using qPCR. The results showed that shPIAS2_49 transfection did not induce endogenous *CRX* or *NRL* mRNA expression (Fig 3A and 3B). On the contrary, when shPIAS2_49 was co-transfected with the CRX and NRL expression vectors, the expression level of both *CRX* and *NRL* mRNA was decreased to about 50%. These results suggest that shRNA does not regulate rho-Gluc activity by mediating *CRX* and *NRL* mRNA expression. We also examined whether rho-Gluc activating shRNAs alter the expression level of endogenous PIAS3, which could result in increased NRL activity. However, no significant change in the endogenous *PIAS3* mRNA expression level was detected (Fig 3C).

**Fig 4:**
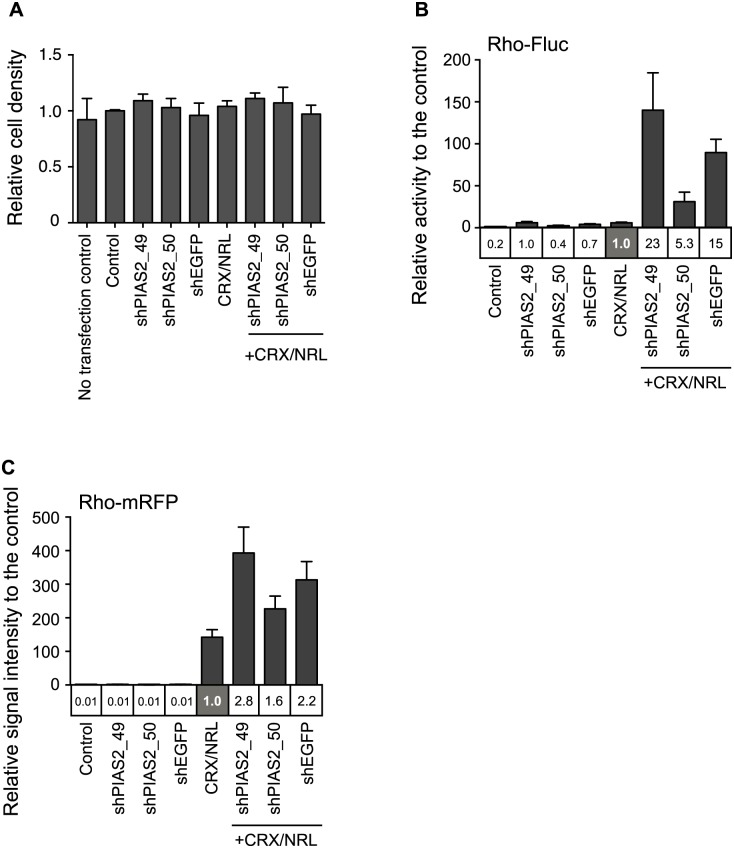
shRNAs do not regulate reporter activity by mediating CRX/NRL expression, cell density, and reporter stability. (A) Relative cell density for shRNA transfected cells. HEK293 cells were transfected with indicated shRNAs in combination with CRX, and NRL expression vectors. Two days after the transfection, the nuclei of the cells were stained with Hoechst 33342 and imaged with the Cellomics Vti High Content Imaging system. The cell density was quantified using Target Activation BioApplication. Relative cell density was presented as the ratio to the cell density of the control. (B, C) The effect of shRNA on rho-Fluc (B) and rho-mRFP (C) reporters. HEK293 cells were transfected with the reporter and the indicated shRNAs with or without CRX and NRL expression vectors. As a control, 500 ng of empty pcDNA3 vector with 100 ng reporter vector was transfected into HEK293 cells. Two days after the transfection, *Firefly* luciferase activity and mRFP fluorescent intensity were measured. Relative luciferase activity to the control (bar graph) and the CRX/NRL transfected sample (in the column under the bar graph) are presented. Error bars are shown as SE.

We next examined if shRNA transfection causes shRNA species-dependent cytotoxicity. Since shPIAS2_49 and shEGFP affect CMV, SV40, and TK promoter-driven reporter activity (data not shown), we did not transfect an internal control reporter for normalization. Therefore, differences in live cell number due to plasmid-dependent cytotoxicity can affect the relative level of rho-Gluc activity measured. Two days post-transfection, cell nuclei were stained with Hoechst 33342. Images were captured and analyzed to determine cell density using a Cellomics Vti Image analysis system. We did not observe a significant change in the cell density between the various transfected cultures (Fig 4A). We also saw no significant difference in cell density between transfected and non-transfected cells under the conditions used suggesting that variable cytotoxicity does not explain the differences in the measured rho-Gluc activity we are measuring.

*Gaussia* luciferase is derived from *Gaussia princeps* and is one of the brightest luciferases known [21]. It catalyzes the oxidation of the substrate coelenterazine without ATP to generate bioluminescence. The secreted *Gaussia* luciferase is stable in culture medium at 37°C for over 7 days (https://www.neb.com/products/e3300-biolux-gaussia-luciferase-assay-kit). The shRNAs that enhance the Gaussia rhodopsin promoter reporter may somehow affect a pathway that interferes with the biochemical properties and/or stability of *Gaussia* luciferase, which could in turn alter the reporter activity in an unexpected fashion. We addressed this possibility by changing the reporter to *Firefly* luciferase (Fluc), which has different reporter chemistry and is one of the first generation luciferases that catalyze the oxidation of luciferin in two-steps in the presence of ATP to yield light. We found that shPIAS2_49 and shEGFP induced rho-Fluc activity and synergistically enhanced the activity in the presence of CRX and NRL (Fig 4B), while shPIAS2_50 had minimal effect on rho-Fluc activity compared with shPAIS2_49 and shEGFP; a similar phenomenon that we observed using Gluc. We also used a fluorescent protein mRFP as a reporter. Similar to the luciferase reporters, shPAIS2_49 and shEGFP synergistically enhanced rho-mRFP signal intensity in the presence of CRX and NRL, while shPIAS2_50 had a much weaker effect on the signal intensity (Fig 4C). We could not observe any alteration in rho-mRFP signal intensity by shRNA itself. This may due to lower sensitivity of the fluorescent reporter. Thus, the shRNA effect on *rho* promoter driven expression was seemingly independent of the reporter used to measure the activation of expression, suggesting that certain shRNAs may affect a pathway that stimulates promoter activity.

### shRNAs activate other promoters

**Fig 5:**
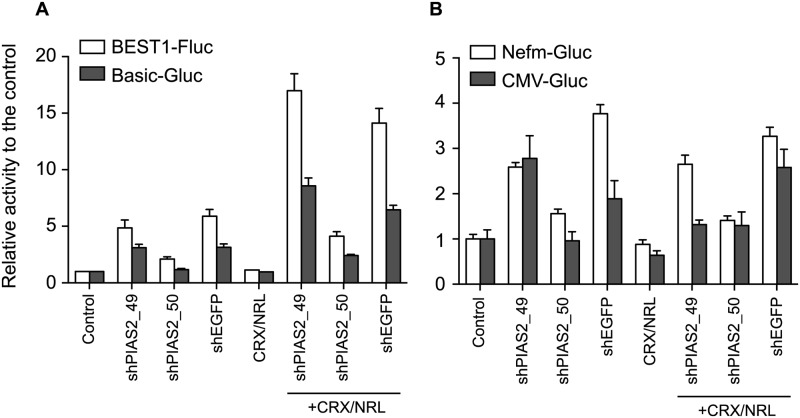
shRNA regulates the activity of the other promoter reporters. HEK293 cells were co-transfected with the indicated shRNAs with or without CRX and NRL expression vectors in the presence of BEST1-Fluc, Basic-Gluc (both in A), Nefm-Gluc, or CMV-Gluc (both in B). Two days after the transfection, *Firefly* and *Gaussia* luciferase activities were measured. As a control, 500 ng of empty pcDNA3 vector with 100 ng reporter vector was transfected into HEK293 cells. Relative activity was presented as the ratio to the control. Error bars are shown as SE.

We next asked if the shRNAs that are activating the *rho* promoter in a seemingly target-agnostic manner can also affect the activity of other promoters. Four promoter reporter constructs (Nefm-Gluc, CMV-Gluc, pGLuc-Basic (designated as Basic-Gluc), and BEST1-Fluc) were prepared. Each reporter was transfected into HEK293 cells in combination with the CRX and NRL expression vectors and shRNA. Except for the *BEST1* promoter, the promoter region used does not contain known or predicted CRX and NRL binding sites. With the BEST1-Fluc and Basic-Gluc reporters, shPIAS2_49 and shEGFP induced the promoter activity by themselves and synergistically enhanced the promoter activity in the presence of CRX and NRL even though Basic-Gluc is a promoter-less reporter. On the other hand, shPIAS2_50 had weaker effect on these promoter activities (Fig 5A). Thus, the shRNAs had a similar effect on these promoters as they did with the rho-Gluc reporter. With Nefm- and CMV-Gluc reporters, shPIAS2_49 and shEGFP induced the promoter activity by themselves while shPIAS2_50 had weaker effect on the promoter activity (Fig 5B). Co-transfection of the shRNA with the CRX and NRL expression vectors did not significantly affect the promoter activities. These results suggest that certain species of shRNA can promote transcription of various reporter constructs in a promoter-independent fashion, suggesting perhaps a more general effect on transcription mechanism(s).

### shRNA sequence and rho-Gluc activity

**Fig 6:**
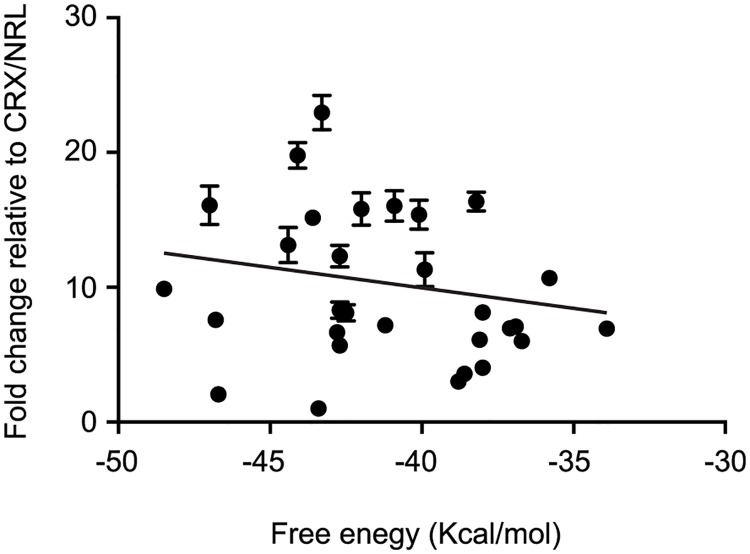
shRNA-mediated rho-Gluc activity and thermodynamic stability. The minimum free energy of shRNAs and their effect on rho-Gluc activity were plotted. Error bars are shown as SE.

Although the shRNA-mediated activation of rho-Gluc activity is target-gene-independent, it is shRNA sequence-dependent. Given the fact that thermodynamic stability of an shRNA species can influence loading of the double stranded RNA into the RNA-induced silencing complex (RISC) [22], variability in the degree of the off-target effect among shRNAs may depend on the loading efficiency of the shRNAs into the RISC. We predicted minimum free energy of the stem-loop structure of shRNAs by CentroidFold (http://www.ncrna.org/centroidfold), and examined if there is a correlation between the thermodynamic stability of the shRNAs and their effect on rho-Gluc activity. Using shRNAs whose minimum free energy ranges from -48.5 to -33.9 kcal/mol (Table 1), we found only a poor correlation between these two factors (R^2^ = 0.051) (Fig 6), suggesting that the thermodynamic stability is not responsible for this shRNA off-target effect.

### shRNA effect on the reporter activity is cell line-dependent

**Fig 7:**
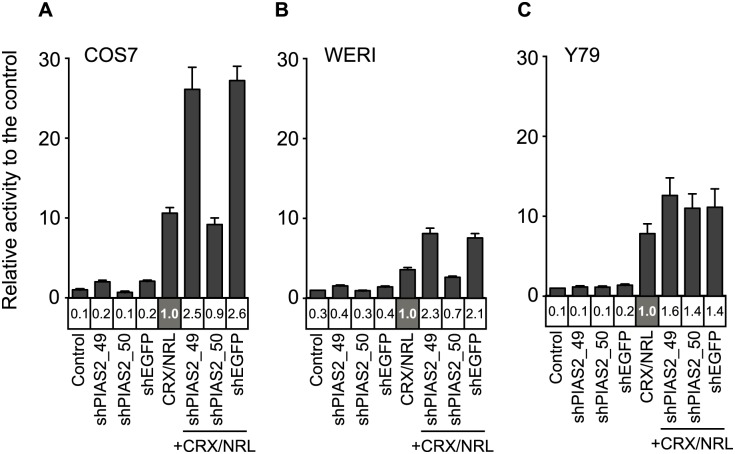
shRNA effect on rho-Gluc activity varies depending on the cell lines transfected. Transfection assay with rho-Gluc reporter and the indicated shRNAs with or without CRX and NRL expression vectors was performed in COS-7 (A), WERI (B), and Y-79 (C) cells. As a control, 500 ng of empty pcDNA3 vector with 100 ng reporter vector was transfected into the cells. Culture medium was collected two days after the transfection to measure *Gaussia* luciferase activity. Relative luciferase activity to the control (bar graph) and the CRX/NRL transfected sample (in the column under the bar graph) are presented. Error bars are shown as SE.

We next wanted to determine whether the observation that certain shRNAs effect transcriptional activation of reporter constructs in a nonspecific manner was a cell-type specific phenomenon. We transfected the rho-Gluc reporter with either shEGFP, shPIAS2_49 or shPIAS2_50, with and without the CRX and NRL expression vectors, into monkey kidney (COS7) and two human retinoblastoma cell lines (WERI-Rb, Y79). In COS7 and WERI cell lines, the shRNA effects on rho-Gluc activity were consistent with those observed in HEK293 cells: while shPIAS2_49 and shEGFP stimulated rho-Gluc activity, shPIAS2_50 did not increase rho-Gluc activity (Fig 7A and 7B). Interestingly, in Y79, unlike WERI and COS7, the effect of shPIAS2_49 and shEGFP on rho-Gluc activity was as low as that of shPIAS2_50 (Fig 7C). None of the shRNA species tested increased rho-Gluc activity by themselves and activity was enhanced only by 40–60% in the presence of CRX and NRL. There was no significant difference between the shRNAs in terms of their effect on rho-Gluc activity. Thus, the observed gene-target-independent effect on rho-Gluc activity is cell line-dependent, suggesting that unidentified endogenous factors that exist in HEK293, COS7, and WERI cells, but not in Y79 cells, may be involved in the shRNA promoter effect.

### Endogenous miRNA expression is altered by shRNA transfection

Transfected pre-shRNAs mature via the miRNA biogenesis pathway, potentially overwhelming the pathway and affect endogenous miRNA processing and maturation [23, 24]. Recent reports suggest that miRNAs can regulate gene transcription by post-transcriptionally suppressing the expression level of certain transcription factors [25]. Disrupted miRNA expression, resulting in altered gene transcriptional activity, thus seemed to be a potential mechanism to account for some of the complex shRNA effects that we were observing.

**Fig 8:**
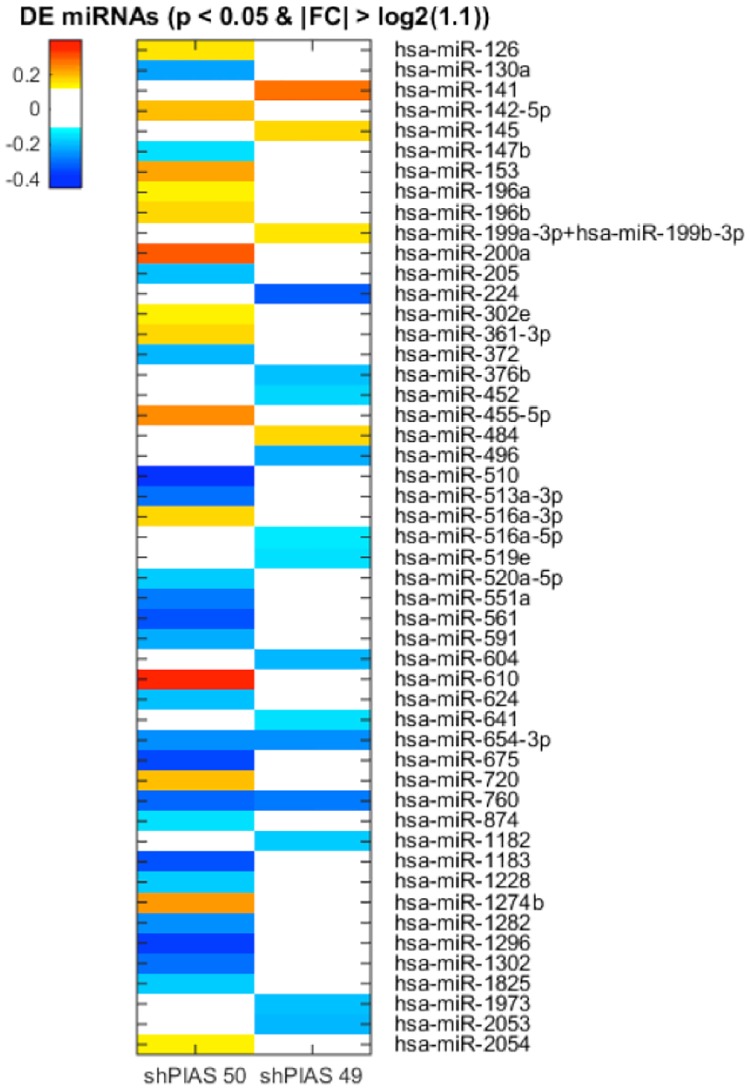
shRNA transfection causes alteration of endogenous miRNA expression. HEK293 cells were transfected with the CRX and NRL expression vectors with or without shPIAS2_49 or shPIAS2_50. Cells were collected at 15, 24, and 48 hrs post-transfection and used for miRNA expression analysis as described in the Materials and Methods. The figure shows miRNAs whose expression was differentially expressed by shPIAS2 transfection. The color represents the fold change (FC) in logarithmic scale (base 2).

To test this possibility, we transfected the CRX and NRL expression vectors with or without shPIAS2_49 or shPIAS2_50 and identified miRNAs whose expression level was altered by the shRNA transfection. We found that 541 miRNA species are expressed in HEK293 cells. Among these, 14 miRNA species were upregulated by shPIAS2_50 co-transfection with the CRX and NRL expression vectors compared to the CRX and NRL transfected cells, while 21 miRNA species were downregulated (paired t-test, p < 0.05, linear |FC| > 1.1). On the other hand, 4 miRNA species were upregulated by shPIAS2_49 co-transfection with the CRX and NRL expression vectors compared to the CRX and NRL transfected cells, while 13 miRNA species were downregulated (p < 0.05, linear |FC| > 1.1). The expression level of 2 miRNA species, hsa-miR-654-3p and hsa-miR-760, was affected by both shPIAS2_50 and shPIAS2_49 transfection, and they were both downregulated (Fig 8). Our results show that shRNA transfection indeed affects endogenous miRNA expression in HEK293 cells, and that the miRNA species that are affected vary depending on the transfected shRNA sequence. The differential expression of miRNA associated with shPIAS2_49 may suggest a possible mechanism by which this shRNA species enhances promoter reporter expression.

### Endogenous gene expression is altered by shRNA transfection

**Fig 9:**
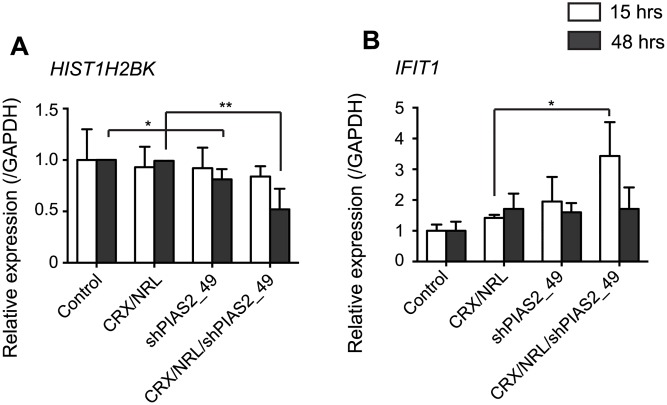
shRNA transfection causes alteration of endogenous protein-coding gene expression. HEK293 cells were transfected with the indicated expression vectors and rho-Gluc reporter. Fifteen- and forty-eight-hour post-transfection, cells were collected for qPCR verification of microarray results. QPCR was performed for the selected genes, *HIST1H2BK* (A) and *IFIT* (B), identified by microarray. The relative expression level was normalized by that of *GAPDH* and calculated as a ratio to the control sample. *P < 0.05, **P < 0.01.

In addition to determining differential expression of miRNAs, we conducted microarray analysis to identify protein-coding genes whose expression level is altered by shPIAS2_49 transfection in HEK293 cells. We first determined differentially expressed genes in shPIAS2_49 transfected cells. Within the 320 genes differentially expressed when cells were co-transfected with shPIAS_49 with CRX and NRL compared to cells just transfected with CRX and NRL (p < 0.1, linear |FC| > 1.25) (S1 Table), 103 genes were upregulated, and 217 genes were downregulated. Gene ontology (GO) functions were not significantly over-represented in the up-regulated gene group, while GO functions that were significantly enriched in down-regulated gene group were “heterocyclic compound binding” and “nucleobase-containing compound metabolic process”. Thus, the majority of the differentially expressed genes do not seem to be directly related to gene transcription. The most predominant gene family that was differentially expressed was the histone gene family (*HIST1H4C*, *HIST1H4E*, *HIST1H4I*, *HIST1H2AE*, *HIST1H2BJ*, *HIST1H2BK*, and *HIST2H2AB*). They consist of 2.19% of the differentially expressed genes (p = 1.2×10^−4^) and all of them were downregulated. QPCR also verified that the expression of *HIST1H2BK*, one of the most predominantly affected genes, was significantly suppressed by shPIAS2_49 transfection (Fig 9A). In addition to histone genes, several interferon response genes were identified as being differentially expressed. Interferon response is a well-known mechanism that can be induced by transfecting double-stranded RNAs and it can cause off-target effects [26–29]. QPCR verified that *IFIT1* mRNA expression was significantly increased by shPIAS2_49 transfection at 15 hrs post-transfection (shPIAS2_49/CRX/NRL vs. CRX/NRL) (Fig 9B).

Finally, we asked if there were potential targets of the identified-miRNAs in the aforementioned assay among the set of differentially expressed genes. Three of them (*HIST1H2AE*, *HIST1H2BJ*, and *HIST2H2AB*) were predicted targets of hsa-miR-760, whose expression was altered by both shPIAS2_49 and shPIAS2_50 transfection (TargetScan, http://www.targetscan.org). We found that two upregulated genes, *ST8SIA2* and *ZNF532*, were the predicted targets of hsa-miR-452, whose expression was specifically downregulated by shPIAS2_49 transfection. Five other differentially expressed genes, *CCNT2*, *MXD1*, *PKN2*, *RANBP9*, and *SKP1*, were also predicted targets of hsa-miR-452, but their expression levels were downregulated by the PIAS2_49 transfection. Interferon genes identified were not potential targets of the identified-miRNAs. Thus, we have identified genes whose expression level is affected by shPIAS2_49 transfection. Further study to investigate if they are involved in the regulation of promoter activity could help elucidate the shRNA off-target regulatory mechanism.

## Discussion

shRNAs are convenient and widely used tools to study the functions of genes of interest by silencing target mRNA expressions (gene knockdown, i.e. loss-of-function). Through use of this approach, we initiated this study with the goal of exploring the potential role of PIAS2 in modulating rhodopsin gene expression. We found that some of the *PIAS2* shRNAs tested demonstrated strong stimulation of rho promoter activity, which at first suggested that PIAS2 could act as a negative regulator of rhodopsin gene expression. However, we noted the potentially problematic finding that there was no correlation between the degree of PIAS2 knockdown effect and the degree of rho promoter activity. Through follow-up studies, we found that the observed shRNA-induced activation of rho-Gluc activity was likely due to off-target effects, mediated in a target gene-independent but highly shRNA sequence-specific manner, a finding that emphasizes the importance of proper controls in shRNA and other RNAi-based studies. Significantly, given that similar shRNA effects on the promoter activity were observed with several other promoter reporters derived from different genes, the observed off-target effect is not rhodopsin promoter-specific. It is also not reporter-specific. Indicating additional complexity, our findings also show that the degree of the shRNA off-target effect can vary by cell-type.

There are several potential pathways that could be responsible for the paradoxical shRNA effects on promoter activity that we observed. Considering that shRNAs take advantage of the endogenous RNAi system to be processed and associated with their target mRNAs, one possible hypothesis is that shRNA expression hinders proper miRNA biogenesis and function, causing mis-regulation miRNA target gene expression, in turn leading in turn to upregulation of promoter activity. In fact, previous studies have shown that competitive inhibition of the endogenous small non-coding RNA processing mechanism can occur due to shRNA over-loading, resulting in cell-death [23, 30] and abnormal spermatogenesis [31]. Supporting this hypothesis, our nCounter miRNA expression assay shows that miRNA expression level is indeed altered by shRNA transfection. Interestingly, miRNAs are differentially expressed by shRNA transfection in an shRNA sequence-dependent manner: 17 and 35 miRNA species are differentially expressed by shPIAS2_49 and _50 transfection, respectively, and only 2 of them are regulated by both shPIAS2_49 and _50. Since it is likely that each shRNA shares the common RNAi processing pathway to produce mature double-stranded siRNAs, shRNAs may disrupt miRNA expression not by over-loading as shown in the previous reports but by an uncharacterized siRNA strand-dependent mechanism in the RNAi system.

Based on our identification of miRNAs whose expression was specifically altered by shPIAS2_49 transfection, which enhanced rhodopsin promoter activity, we next investigated protein-coding genes whose expression was differentially altered by shPIAS2_49 transfection, and which were potential targets of the identified miRNAs. By microarray analysis of differentially expressed genes followed by computational analysis of potential miRNA target sites at the 3'-end of the differentially expressed genes, we tried to identify the genes that could be responsible for the off-target effect. Among the genes identified, histone genes were the most predominant class (2.19% of the differentially expressed genes) and three of them (*HIST1H2AE*, *HIST1H2BJ*, and *HIST2H2AB*) are potential targets of hsa-miR-760, whose expression was altered by shPIAS2_49. It is unclear how modulating histone mRNA may directly influence promoter activity; it seems unlikely that potential changes in chromatin structure would affect accessibility of a promoter sequence on a naked, histone-less plasmid. However, since the shRNA-associated activation of promoters seems to be a general phenomenon in certain cell lines (i.e. not promoter specific), one possibility is that a very general cellular process that affects transcription and/or transcriptional timing could be affected by these shRNA species, and the change in histone mRNA expression may be a reflection of this affect.

Another plausible pathway that is affected by the shRNAs that influence promoter activation in a target-independent manner is the interferon response pathway [32]. Transfection of long double-stranded RNAs can induce interferon response [33]. This response is minimized by using short (21 nucleotides (nt)) siRNAs [34], but with the vector-based RNAi system, such as shRNAs, shRNA transfection can induce a strong interferon response [26, 27]. In neuronal cells, it has been suggested that the induced interferon response can cause structural and functional cellular abnormalities [26]. In our study, we found that some interferon-stimulated genes are upregulated by shPIAS2_49, consistent with the possibility that the interferon response may, at least in part, be responsible for the observed upregulation of promoter activity. However, further research will be required to elucidate if and how the interferon response is involved in the shRNA off-target effects such as those described in this paper.

In summary, although shRNAs can be powerful tools for manipulating gene expression in a target-specific manner [35], they can also have unexpected off-target effects that can affect a variety of cellular phenotypes. As just a few of the examples reported in the literature, off-target effects can be responsible for cytotoxicity [23, 24, 30], defects in synaptogenesis [26], neuronal migration [36], spermatogenesis [31], and papillomavirus positive cervical cancer cell death [37]. In our work described here, we add to this long list of potential unwanted shRNA effects, showing that shRNA off-target effects can inadvertently influence transcription reporter assays in strong, complex, misleading, and unexplained ways. On the positive side, it should be noted that active efforts are underway to improve the specificity of shRNA technology [38]. McBride et al. developed a miRNA-based expression system, with decreased siRNA expression levels, that resulted in significant reduction of neurotoxicity in the brain [30]. Mockenhaupt et al. demonstrated that off-target effects can be alleviated by co-delivering inhibitory decoy RNAs [39]. Furthermore, Herrera-Carrillo showed that a special shRNA class, termed AgoshRNA, whose hairpin structure is processed directly by Ago2 in a Dicer-independent manner, can eliminate passenger strand-mediated off-target effects [40]. Also, improved shRNA design programs are available [41]. These ongoing improvements, combined with careful experimental design, and attention to appropriate controls, will hopefully help in the field’s continuing efforts to avoid misleading conclusions.

## Supporting Information

S1 Table List of genes whose expressions are altered by shRNA transfection. HEK293 cells were transfected with the CRX and NRL expression vectors with or without shPIAS2_49. Fifteen and forty-eight hours post-transfection, the cells were collected to extract total RNA. Microarray analysis was performed as described in the Materials and Methods. DEG, differentially expressed gene; FC, fold change.
(XLSX)
